# The clinical and imaging features of cerebrotendinous xanthomatosis: A case report and review of the literature

**DOI:** 10.1097/MD.0000000000024687

**Published:** 2021-03-05

**Authors:** Chi Ma, Yan-De Ren, Jia-Chen Wang, Cheng-Jian Wang, Ji-Ping Zhao, Tong Zhou, Hua-Wei Su

**Affiliations:** Department of Radiology, Affiliated Hospital of Qingdao University, Qingdao, Shandong, China.

**Keywords:** cerebrotendinous xanthomatosis, dentate nucleus, magnetic resonance imaging

## Abstract

**Rationale:**

Cerebrotendinous xanthomatosis (CTX) is a rare autosomal recessive lipid deposition disorder characterized by systemic signs and neurological dysfunction. The radiological features of CTX are infrequently summarized in the literature.

**Patient concerns:**

We described a 40-year-old male patient who repeatedly engaged in wrestling matches and presented with progressive difficulty in walking and reduced balance with egg-sized, hard, smooth, and painless masses in both ankles.

**Diagnosis:**

Neuroimaging examination showed abnormalities both supra- and infratentorially. Bilateral ankle joint magnetic resonance imaging showed bilateral xanthomata of the Achilles tendon. The diagnosis was confirmed by the detection of a sterol 27-hydroxylase gene mutation.

**Interventions:**

The patient was treated with chenodeoxycholic acid (250 mg 3 times per day).

**Outcomes:**

To date, the patient's bilateral xanthomas of the Achilles tendon have begun to diminish, and his neurological impairment has not deteriorated further but has not yet improved.

**Lessons:**

We report a rare case of CTX and summarize the clinical and imaging features of this disease. Our findings suggest that the abnormal signals in the dentate nucleus or a long spinal cord lesion involving the central and posterior cord, combined with tendon xanthoma, are important clues for the diagnosis of CTX.

## 1 Introduction

Cerebrotendinous xanthomatosis (CTX) is a rare autosomal recessive lipid deposition disorder caused by a sterol 27-hydroxylase (CYP27A1) gene mutation. CYP27A1 is a widely expressed mitochondrial enzyme belonging to the mitochondrial chrome P450 enzyme family that is responsible for catalyzing multiple hydroxylation reactions in cholesterol metabolism and bile acid synthesis. CYP27A1 deficiency leads to reduced production of chenodeoxycholic acid (CDCA) and the accumulation of cholestanol and cholesterol in many tissues, especially in the brain, lens, and tendons.^[1,2]^ There is marked heterogeneity of signs and symptoms in CTX, and early diagnosis and treatment are crucial to prevent the progression of neurological dysfunction.^[3]^ Radiological features of the disease are infrequently discussed in the literature. It is important to recognize these abnormalities because the progression of MR abnormalities is related to treatment effectiveness and the prognosis of patients.^[4]^ The aim of the present study is to report a case of CTX and provide a literature review on the clinical and imaging features and possible pathological mechanisms of previously reported cases of CTX.

## 2 Case report

A 40-year-old man experienced a 2-year history of progressive difficulty with walking, reduced balance, and repeated engagement in wrestling matches. At the age of 38, he was misdiagnosed with “right lower extremity erysipelas” due to sudden lower extremity weakness and painful swelling along the posterior area of his right ankle joint, and the symptoms improved after treatment. Nearly 6 months earlier, his gait began to deteriorate, he could not perform tandem gait smoothly and he was unable to walk without assistance. He had normal development and did not have a history of neonatal jaundice, cataracts, or infantile diarrhea. He finished elementary middle school. He was born to nonconsanguineous parents, and there was no significant family history.

Physical examination demonstrated bilateral egg-sized, hard, smooth, and painless masses in the Achilles tendons. Neurological examination showed mild muscle hypertonia, and deep tendon reflexes were mildly enhanced bilaterally with positive Babinski reflexes and ankle clonus, indicating impairment in the bilateral pyramidal tracts. He showed mild cognitive and language impairment. His laboratory tests showed no abnormalities, including liver and kidney function, serum electrolytes, triglyceride, and cholesterol levels. An electroencephalography examination revealed slow background activity composed of theta and delta waves, occasionally accompanied by high-voltage activity.

**Figure 1:**
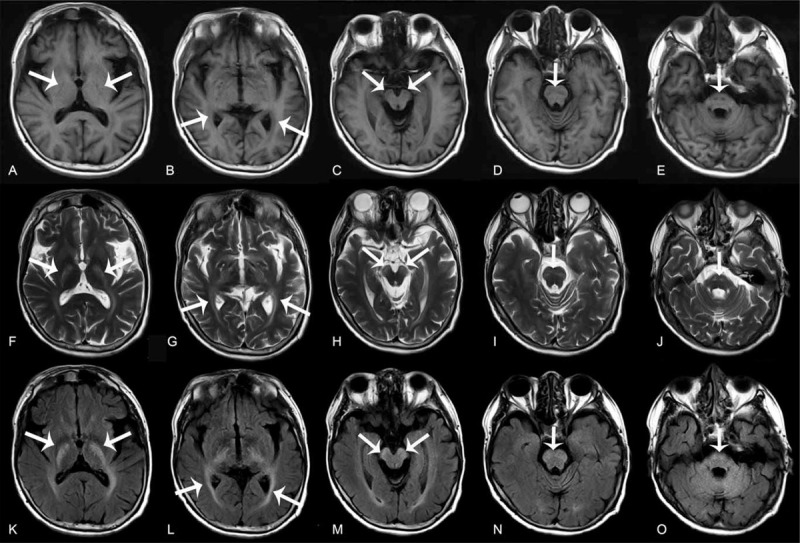
Brain abnormality detected by MRI in the patient. (A) to (E) Axial T1W, (F) to (J) T2W, and (K) to (O) FLAIR images show symmetric hyperintensities in the posterior limbs of internal capsules, paraventricular white matter, cerebral peduncles and anterior region of the pons on T2W and FLAIR images (arrows) with corresponding hypointensities on T1W images, which are along corticospinal tracts. FLAIR = fluid-attenuated inversion recover, T1W = T1-weighted, T2W = T2-weighted.

**Figure 2:**
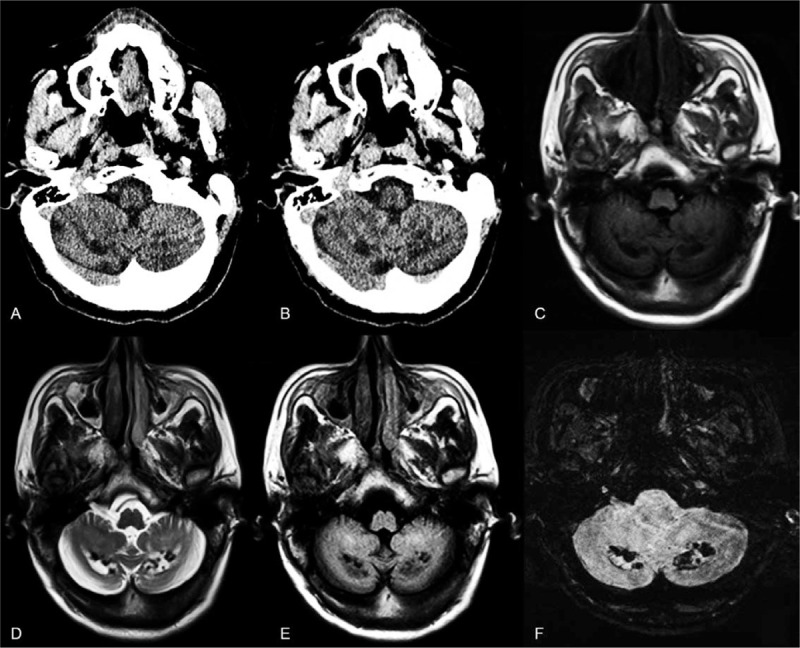
Cerebellar parenchymal abnormalities in the patient. (A) and (B) Axial CT scans show low density in both cerebellar hemispheres. (C) Axial T1W images show hypointensities in both dentate nuclei and bilateral cerebellar deep white matter. (D) Axial T2W images show hyperintensities in the bilateral cerebellar deep white matter with low-intensity areas in the dentate nuclei. (E) Axial FLAIR image shows low signal intensity consistent with cerebellar vacuolation in the same areas. (F) SWI reveals hypointensities in the bilateral dentate nuclei compatible with microcalcifications. CT = computed tomography, FLAIR = fluid-attenuated inversion recovery, SWI = susceptibility-weighted imaging, T1W = T1-weighted, T2W = T2-weighted.

**Figure 3:**
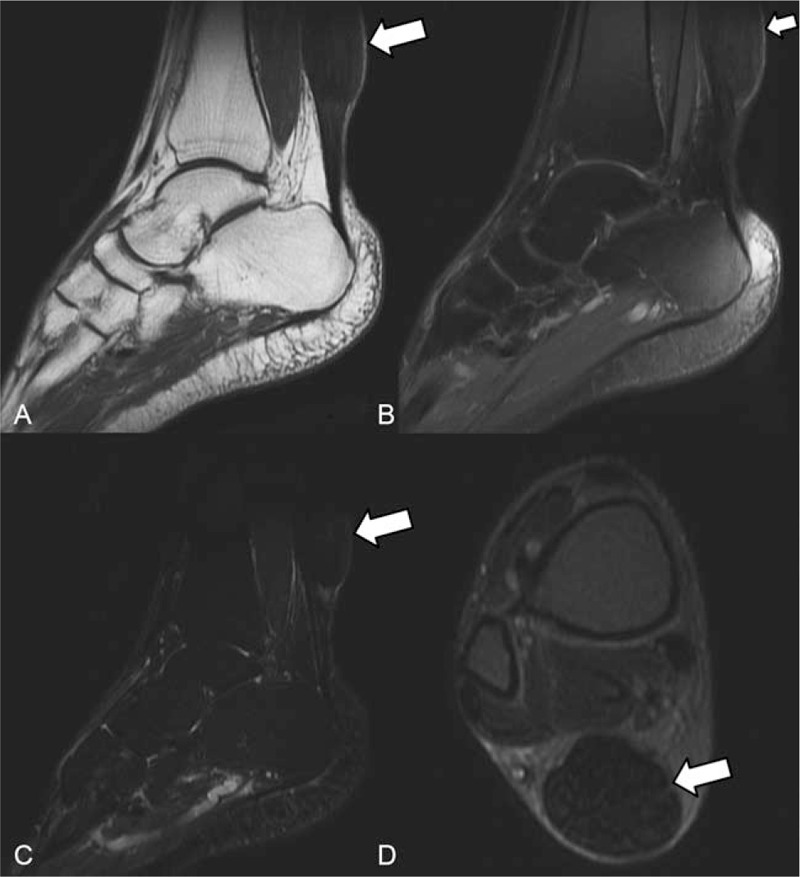
Achilles tendon xanthoma (right) in the patient. (A) Sagittal T1W, (B) proton density, and (C) STIR images show fusiform enlargement of the right Achilles tendon (arrows); the xanthoma is relatively isointense compared to muscle. (D) An axial proton density image shows diffuse fat infiltration with low-intensity tendon bundles interspersed within (arrows). STIR = short tau inversion recovery, T1W = T1-weighted.

We performed brain magnetic resonance imaging (MRI) that revealed mild cerebral and cerebellar atrophy, low-intensity areas in the dentate nuclei, and symmetric hyperintensities in the cerebellar deep white matter, cerebral peduncles, anterior region of the pons, posterior limbs of internal capsules, and paraventricular white matter on T2-weighted (T2W) and fluid-attenuated inversion recovery (FLAIR) images with corresponding hypointensities on T1-weighted (T1W) images (Fig. 1 and Fig. 2C-E). Susceptibility-weighted imaging (SWI) revealed hypointensities in the bilateral dentate nuclei (Fig. 2F). Brain computed tomography (CT) scans showed low density in both cerebellar hemispheres (Fig. 2A and B). Bilateral ankle joint MRI showed fusiform enlargement of the Achilles tendon, which was isointense to the muscle (Fig. 3). Heredity metabolic disease was suspected at this point, and the diagnosis was confirmed by demonstrating the mutation of the CYP27A1 gene. CDCA treatment (250 mg 3 times per day) was initiated for the patient; more than 3 months later, his bilateral xanthomas of the Achilles tendons began to diminish, but no improvement in his neurological signs was observed.

## 3 Discussion

CTX progresses slowly and has a clear variability in clinical presentation and onset age. Progressive neurological dysfunction, adolescent cataract, tendon xanthoma, and chronic diarrhea are the most common clinical manifestations. Premature atherosclerosis, cardiovascular disease, neonatal cholestatic jaundice, osteoporosis with repeated bone fractures, and pulmonary symptoms have also been reported in some patients with CTX.^[1,2]^

To date, more than 300 cases of CTX have been reported in the English literature search on PubMed. We only summarized the clinical (Table 1) and imaging (Table 2) features of published literature from 2016 to 2019^[5–25]^ and excluded papers without clinical case descriptions and detailed imaging data. There were 25 patients from 22 families, including the current case. The average age of these patients was 36.6 years (range 8 months–77 years, median 38 years), and the female-male ratio was 11:14. Neurological symptoms were the most common clinical manifestations in 92% (23/25) of the patients, including intellectual disability, cognitive impairment, dementia, gait disturbance, psychiatric symptoms (eg, depression, schizophrenia, anxiety, and impatience), pyramidal signs (eg, weakness, hyperreflexia, and spasticity), cerebellar signs (eg, ataxia, dysarthria, and nystagmus), peripheral neuropathy, seizures, and parkinsonism. Other rarely reported neuropathies included oculomotor apraxia and optic neuritis. The occurrence of bilateral cataracts was reported in 60% (15/25) of the patients, tendon xanthoma was reported in 68% (17/25) of the patients, and only 20% (5/25) of the patients reported chronic diarrhea. Both osteoporosis, saccular-type abdominal aortic aneurysm and neonatal jaundice were reported in only 4% (1/25) of cases. Tendon xanthomas were most commonly located in bilateral ankles, followed by the knees, elbow, fingers, tibia, and other locations. Bilateral cataracts, mental retardation, and chronic diarrhea seem to be the earliest symptoms in childhood, but these symptoms were often overlooked until neurological symptoms occurred, leading to a delay in diagnosis that may be fatal.

**Table 1:** Summary of clinical features in previously reported CTX patients.

			Clinical features						
Case	Author/year	Age/sex	Neurological symptom	Cataracts	Tendon xanthoma	Diarrhea	Osteoporosis/juvenile bone fractures	Premature atherosclerosis and cardiovascular disease	Neonatal cholestatic jaundice
1	Parry et al^[5]^/2019	32/F	+	+	Bilateral ankles, right malar emminence	−	−	−	−
2	Lee et al.^[6]^/2019	38/M	+	−	Bilateral ankles	−	−	−	−
3		37/M	+	−	Bilateral ankles, knees, and elbows	−	−	−	−
4	Shaji et al^[7]^/2019	35/M	+	+	Bilateral ankles	−	−	−	−
5	Miyamoto et al^[8]^ /2019	39/M	+	+	Bilateral ankles, knees, and triceps	−	−	−	−
6	Mutlu et al^[9]^/2019	42/F	+	−	−	−	−	−	−
7	Gelzo et al^[10]^/2019	28/F	+	+	−	+	−	−	−
8	Tada et al^[11]^/2018	63/M	−	−	Bilateral ankles and plantar	−	−	Saccular type abdominal aortic aneurysm	−
9	Shen and Wang^[12]^/2018	8month/F	+	−	−	−	−	−	+
10	Weissfeld et al^[13]^/2018	45/F	+	+	−	−	−	−	−
11	Sasamura et al^[14]^/2018	50/F	+	+	Bilateral ankles and right triceps	−	+	−	−
12	Nambirajan et al^[15]^/2018	23/F	+	+	Bilateral ankles	+	−	−	−
13		25/F	+	+	Bilateral ankles	+	−	−	−
14	Mukaino et al^[16]^/2018	47/M	+	+	Bilateral ankles and the left tibia	+	−	−	
15	Zadori et al^[17]^/2017	40/F	+	+	−	+	−	−	−
16		40/F	+	+	−	−	−	−	−
17	Abdel-Hamid et al^[18]^/2017	35/M	+	−	Elbows, knees, and ankles	−	−	−	−
18	Alhariri et al^[19]^/2017	36/M	+	−	Right triceps and bilateral ankles	−	−	−	−
19	Gerrish and Gaba^[20]^/2017	63/M	+	+	Right finger, right ankle	−	−	−	−
20	Razi et al.^[21]^/2016	25/F	−	+	Ankles and both knees	−	−	−	−
21	Abe et al^[22]^/2016	46/M	+	−	−	−	−	−	−
22	Yanagihashi et al^[23]^/2016	77/M	+	−	Bilateral ankles and knees	−	−	−	−
23	Kulkarni et al^[24]^/2016	10/M	+	+	−	−	−	−	−
24	Parente et al^[25]^/2016	38/M	+	+	Left ankle	+	−	−	−
25	Current study	40/M	+	−	Bilateral ankles	−	−	−	−

**Table 2:** Summary of imaging features in previously reported CTX patient.

			MRI features (affected region)		
Case	Author/year	Age/sex	Brain	Spine	MRS
1	Parry et al^[5]^/2019	32/F	Dentate nuclei, deep cerebellar white matter, posterior limbs of internal capsules	Normal	NA
2	Lee et al^[6]^/2019	38/M	Dentate nuclei, pons, cerebral peduncles, and periventricular white matter	Normal	NA
3		37/M	Dentate nuclei and cerebellar hemispheres, pons, cerebral peduncles, and periventricular white matter	Normal	NA
4	Shaji et al^[7]^/2019	35/M	Cerebellar atrophy, abnormal signals in the dentate nuclei, globus pallidus, substantia nigra, and periventricular white matter	Normal	NA
5	Miyamoto et al^[8]^ /2019	39/M	Enlarged fourth ventricle, cerebellum atrophy, abnormal signals in the dentate nuclei, and pyramidal tract	Normal	NA
6	Mutlu et al^[9]^/2019	42/F	Internal capsule and crus cerebri and both dentate nuclei	Dorsal columns throughout the cervical and thoracic cord	NA
7	Gelzo et al^[10]^/2019	28/F	The cortico-spinal tracts, deep cerebellar white matter, peripherally located at the dentate nuclei	Cervical spinal cord, the lateral and cortico-spinal tracts of the dorsal columns	NA
8	Tada et al^[11]^/2018	63/M	Periventricular white matter	Normal	NA
9	Shen and Wang^[12]^/2018	8month/F	Normal	Normal	NA
10	Weissfeld et al^[13]^/2018	45/F	Cerebellar dentate nuclei, internal capsule, cerebral peduncles, and posterior periventricular white matter	Normal	NA
11	Sasamura et al^[14]^/2018	50/F	Dentate nuclei, peripheral white matter of the cerebellum, the pyramidal tracts from the pons to the cerebral crus	Normal	NA
12	Nambirajan et al^[15]^/2018	23/F	NA	Normal	NA
13		25/F	Dentate nuclei and adjacent cerebellar white matter	Normal	NAA↓ and the presence of a lactate peak
14	Mukaino et al^[16]^/2018	47/M	Diffuse brain atrophy, abnormal signals in the dentate nuclei, periventricular white matter, and along corticospinal tracts in the posterior internal capsules, cerebral peduncles	Normal	Cho↑and NAA↓
15	Zadori et al^[17]^/2017	40/F	Dentate nuclei and some supratentorial white matter	Normal	NA
16		40/F	Dentate nuclei	Normal	NA
17	Abdel-Hamid et al^[18]^/2017	35/M	Mild cortical and cerebellar atrophy, abnormal signals in the white matter especially around the occipital horn, cerebellum and deep in cerebrum, and dentate nuclei	Normal	NA
18	Alhariri et al^[19]^/2017	36/M	Global cerebral and cerebellar atrophy, abnormal signals in the periventricular white matter and dentate nuclei	The entire cervical and thoracic cord, predominately in the central and posterior cord	NA
19	Gerrish and Gaba^[20]^/2017	63/M	Cerebral and cerebellar atrophy, abnormal signals in the periventricular white matter, dentate nucleus, and the surrounding deep cerebellar white matter	Normal	NA
20	Razi et al^[21]^/2016	25/F	Dentate nucleus	Normal	Cho↑ and NAA↓
21	Abe et al^[22]^/2016	46/M	Normal	Lateral corticospinal tracts and gracile tracts in the cervical and thoracic cord extending from C1 to Th4 level	NA
22	Yanagihashi et al^[23]^/2016	77/M	Normal	Dorsal columns of the C2-C7 spinal cord	NA
23	Kulkarni et al^[24]^/2016	10/M	Cortical and cerebellar atrophy, abnormal signals in the dentate nucleus, and parieto-occipital white matter	Normal	NA
24	Parente et al^[25]^/2016	38/M	Cerebellar and moderate cerebral atrophy, abnormal signals in both middle cerebellar peduncles and dentate nuclei, and progressive calcification was noted in the dentate nuclei after 2 years	The corticospinal and gracile tracts in the cervical cord	NA
25	Present study	40/M	Mild cerebral and cerebellar atrophy, abnormal signals in the dentate nuclei, cerebellar deep white matter, cerebral peduncles, anterior region of the pons, posterior limbs of internal capsules, and bilateral paraventricular white matter	Normal	NA

A diagnosis of CTX is challenging due to its pleiotropic clinical characteristics. Nevertheless, it is doubtless that MRI is one of the key parts of the diagnostic process. Although the MRI findings for CTX have been reported to vary, it might be worthwhile to thoroughly investigate these findings to determine any typical and common MRI features of CTX. A review of the literature has shown brain MRI abnormalities in 84% (21/25) of patients, with cerebral cortical and/or cerebellar atrophy and both supra- and infratentorial signal abnormalities as the main findings. T2W and FLAIR images of the brain showed symmetric hyperintense lesions in the periventricular white matter, posterior limbs of internal capsules, globus pallidum, cerebral peduncles extending into the substantia nigra, anterior region of the pons, inferior olive, or in the cerebellar parenchymal, involving the dentate nuclei and the surrounding white matter, which were hypointense on T1W and diffusion-weighted (DW) images. These signal changes were due to lipid accumulation in nerve cells with subsequent demyelination and axonal degeneration, especially in the latter stages of the disease.^[4,26,27]^ The dentate nuclei may present hypointensities on T2W/FLAIR/SW images over time, such as in our patient, which were reported to be associated with demyelination, hemosiderin deposition, microcalcification, necrosis, or cystic spaces,^[4,27]^ and a study suggested that these signal alterations may be the result of secondary degeneration caused by cholesterol-induced apoptosis or other as-yet-unidentified mechanisms that cause axonal damage and can be regarded as the first available biomarker of disease progression because it can predict clinical and MRI deterioration despite CDCA therapy.^[4]^ T2W/FLAIR signal abnormalities in the dentate nuclei were the most common findings in patients with CTX. The reasons for preferential involvement of the dentate nucleus remain unclear. According to previous reports, the dentate nucleus is vulnerable to neurodegenerative, ischemic, toxic, inflammatory, infectious, or metabolic damage.^[28,29]^ It should be noted that the distribution of the lesions along corticospinal tracts or on the cerebellum were consistent with the clinical presentation of pyramidal or cerebellar signs, and the abnormalities of the substantia nigra may be associated with the Parkinsonian feature. However, brain abnormalities in MRI could be found in patients with no apparent neurological symptoms, suggesting that MRI can detect neurological abnormalities in patients at an early stage, so MR examination is necessary for patients even if they do not exhibit obvious neurological symptoms. There were also spinal form CTX with nonenhancing long T2W hyperintense lesions in 24% (6/25) of patients, which predominately involved the central and posterior cord and had a relatively mild clinical course compared with the classic form of CTX. Magnetic resonance spectroscopy (MRS) revealed typical lipid peaks, increased choline, and decreased N-acetyl aspartate peaks in the involved regions, which indicated extensive axonal damage and mitochondrial dysfunction.^[15,16]^

Tendon xanthomas were inhomogeneously hypo- to isointense on T1W images and showed low to intermediate contrast on T2W images. Bilateral Achilles tendons were most frequently involved. CT scans may show soft tissue enlargement with areas of low attenuation. This may be related to abnormal lipid deposition. Pressure erosion of the bone resulting from these large xanthomas can sometimes be seen on CT scans.^[30]^ The above neuroimaging findings, combined with tendon xanthoma, are important clues for the diagnosis of CTX. In addition, Miyamoto et al^[8]^ reported a case of optic neuritis, presenting as bilateral optic nerve swelling and enhanced MRI images. Other imaging findings included osteoporosis accompanied by pathological fractures and pulmonary interstitial fibrosis,^[2]^ but these were not characteristic manifestations of CTX.

The diagnosis of CTX is mainly based on clinical suspicion, laboratory and imaging findings, and molecular genetic analysis. Diagnosis can be confirmed by the elevation of cholestanol and cholesterol precursor levels in the plasma or cerebrospinal fluid. Serum total cholesterol levels should be normal to low,^[31]^ which contributes to distinguishing CTX from other lipid storage disorders, such as familial hypercholesterolemia (FH). However, our patient's laboratory examination showed no significant abnormalities, so the diagnosis of the disease in our patient was based mainly on clinical and radiological examinations followed by genetic testing. Treatment with CDCA (250 mg 3 times per day) was initiated in our patient. CDCA, standard care for CTX patients, can provide exogenous feedback inhibition of bile acid production as a bile acid replacement and improve the majority of neurologic and other symptoms.^[3]^ After 3 months of treatment, the patient's neurological symptoms did not improve significantly, but bilateral xanthomas of the Achilles tendons began to diminish. Early treatment is essential to reverse neurological symptoms. A recent study by Stelten et al^[32]^ demonstrated that patients who start treatment after age 25 have poorer outcomes than those who start treatment early and may continue to worsen. Moreover, cerebellar microcalcification or cavitation on MRI indicated that the lesion developed from early cholestanol deposition to irreversible apoptosis; it is also possible that we should extend the follow-up time. A retrospective cohort study showed that CDCA was generally effective and acceptable safety, with disease signs and symptoms improved, alleviated, or stabilized in most patients an average of 9.9 months after treatment.^[33]^ Other alternative therapies (such as inhibitors of HMG-CoA reductase,^[34]^ low-density lipoprotein apheresis^[35]^) were considered to have limited efficacy and require more clinical trials.

For the neuroradiologist, the brain MRI abnormalities of CTX should be differentiated from metronidazole-induced encephalopathy (MIE) and Maple syrup urine disease (MSUD). MIE is a rare toxic encephalopathy caused by the commonly used antimicrobial drug metronidazole.^[29]^ Brain MRI of MIE presents symmetrical lesions, without pathologic enhancement, most commonly involving the dentate nucleus, followed by the midbrain, pons, and corpus callosum. The abnormal MRI signal is reversible, and imaging studies return to baseline within a few weeks of metronidazole discontinuation.^[28]^ MIE patients may present cerebellar or psychiatric symptoms but do not present signs of Achilles tendon xanthoma or cataracts. MSUD is an autosomal recessive disorder, and MRI reveals a typical pattern of bilateral symmetric areas involving cerebellar white matter, the dorsal brain stem, bilateral thalami, the globus pallidum, the cerebral peduncles, and internal capsules and corticospinal tracts, with marked restricted diffusion and decreased apparent diffusion coefficient values,^[36]^ which are different from CTX. Spinal form CTX should be differentiated from chronic myelopathies, such as multiple sclerosis (MS) and neuromyelitis optica (NMO). Myelitis in MS is patchy and irregularly distributed rather than symmetrically distributed; it usually has a smaller longitudinal extension (one or two vertebral bodies) and a more peripheral distribution in the spinal cord.^[37]^ NMO is characterized by severe optic neuritis and longitudinally extensive transverse myelitis. NMO spinal lesions are centrally located and preferentially involve gray matter, as it corresponds to the most prominent expression of the aquaporin-4 antigen and extends over 3 or more contiguous segments.^[38]^ Tendon xanthoma has also been observed in other very rare genetic diseases, such as FH and sitosterolemia. However, these two diseases will not have neurological manifestations.

## 4 Conclusion

We report a rare case of CTX along with a literature review on the imaging, clinical features, and possible pathological mechanism of previously reported cases of CTX. Furthermore, we emphasize the importance of imaging examinations. Our findings suggest that the presence of abnormal signals in the dentate nucleus or a long spinal cord lesion involving the central and posterior cord, combined with tendon xanthoma, in the appropriate clinical setting should alert the clinician to the possibility of CTX and the need to screen for mutations in the CYP27A1 gene.

## Author contributions

**Data curation:** Huawei Su, Yande Ren.

**Investigation:** Chi Ma, Jiachen Wang, Chengjian Wang.

**Project administration:** Huawei Su.

**Resources:** Yande Ren.

**Supervision:** Jiping Zhao, Tong Zhou.

**Writing – original draft:** Chi Ma.

**Writing – review & editing:** Huawei Su, Yande Ren.
